# Comparison of conventional sonographic signs and magnetic resonance imaging proton density fat fraction for assessment of hepatic steatosis

**DOI:** 10.1038/s41598-018-26019-x

**Published:** 2018-05-17

**Authors:** Mimi Kim, Bo-Kyeong Kang, Dae Won Jun

**Affiliations:** 1 Department of Radiology, Hanyang University School of Medicine, Seoul, Korea; 2 Department of Internal Medicine, Hanyang University School of Medicine, Seoul, Korea

## Abstract

This study correlated conventional ultrasonography (US) signs with the magnetic resonance imaging (MRI) proton density fat fraction (PDFF) to evaluate the diagnostic performance of US signs (alone or combined) to predict presence and degree of hepatic steatosis (HS). Overall, 182 subjects met the study inclusion criteria between February 2014 and October 2016. Four US signs were evaluated independently by two radiologists. MRI PDFF was defined as the average of 24 non-overlapping regions of interest (ROIs) within eight liver segments obtained by drawing three ROIs within each segment. The latter acted as the reference standard to evaluate diagnostic accuracy of the US signs and their combinations. Diagnostic performance of US for HS was assessed using receiver operating characteristic (ROC) curve analyses. There was a strongly positive correlation between some combinations of US signs and PDFF (σ = 0.780, *p* < 0.001). The sensitivity, specificity, PPV, and NPV were 96.6%, 74.8%, 64.8%, and 97.9%, respectively, determined using abnormal hepatorenal echoes to detect grade 1 or higher HS (area under the ROC curve = 0.875). The sensitivity and NPV for detecting HS with US were good and US may be considered a suitable screening tool for exclusion of HS.

## Introduction

Non-alcoholic fatty liver (NAFLD) is a leading cause of chronic liver disease in most developed countries. The prevalence of NAFLD determined by magnetic resonance spectroscopy and biopsy has been reported to be more than 30% in the general population of the United States^1^. NAFLD affects the progression of chronic liver disorders, but also increases the risk of death by cardiovascular disease^2,3^.

Liver biopsy is the gold standard for the diagnosis of NAFLD. However, liver biopsy is an inappropriate tool for most screening, monitoring, and research because of its invasiveness and other limitations such as sampling error, and interobserver and intraobserver variability^4^. Recently, the proton density fat fraction (PDFF) has been proposed as a noninvasive biomarker of hepatic steatosis (HS)^5–7^. PDFF is defined as the ratio of mobile triglyceride protons to the sum of mobile triglyceride and water protons. Thus, magnetic resonance imaging (MRI)-quantified PDFF (hereafter, MRI PDFF) provides an objective, quantitative estimate of the degree of HS. MRI PDFF of the liver has been shown to have high diagnostic accuracy with histological steatosis grade^8–10^ and has been recognized as the reference standard in diagnostic radiology^11,12^. Determination of MRI PDFF has been developed in various commercial software products [IDEAL IQ (GE), mDixon Quant (Philips), and Multiecho VIBE Dixon (Siemens)]. These have been used instead of liver biopsy in randomized controlled clinical studies^13,14^ and usage is expected to increase in the future.

Although MRI PDFF is used a biomarker of HS, the feasibility of using MRI PDFF in research and clinical settings is limited by the high cost and limited availability. Ultrasound (US), in contrast, is less expensive, widely available, and commonly used in all settings, including research and clinical practice, despite being less accurate and less precise than MRI PDFF^15–18^. Various US signs of HS have been described^19–21^; however, to date, they have not been rigorously compared to MRI PDFF.

Therefore, the purpose of our study was to correlate conventional US signs to MRI PDFF and to evaluate the diagnostic accuracy of each US sign, and their combinations to predict the presence and degree of HS.

## Methods

### Study oversight

We performed a retrospective cohort study at a single academic tertiary hospital. This study was approved by the institutional review board of Hanyang University Hospital and was registered in a clinical trials database (the Korea Clinical Research Information Service, https://cris.nih.go.kr/cris/index.jsp). All experiments were performed in accordance with relevant guidelines and regulations. Informed written consent was obtained from all subjects. MRI PDFF was used as the reference standard of diagnosis and grading of HS.

### Subjects

**Figure 1:**
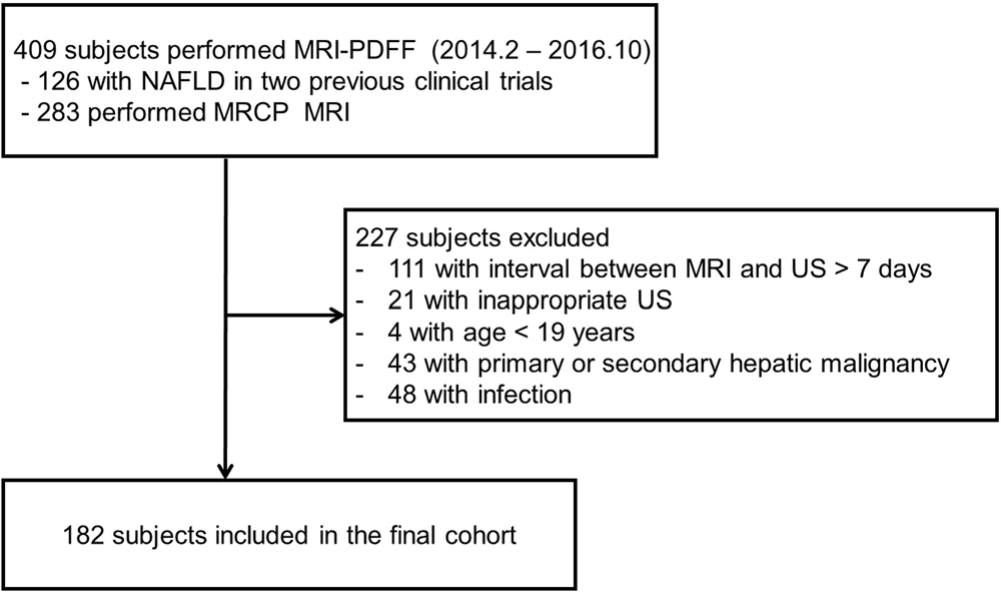
Flow diagram of patient selection.

We included subjects who underwent MRI PDFF between February 2014 and October 2016. First, we enrolled 126 adult subjects with NAFLD who had participated in two previous clinical trials (clinical trial numbers KCT 0001480 and KCT 0001588, https://cris.nih.go.kr/cris/index.jsp) that compared changes of MRI PDFF before and after the use of lactobacillus. For complete details of the inclusion and exclusion criteria of the two parent studies, see Appendix 1. We used baseline MRI PDFF values from the two clinical trials. Next, we included 283 consecutive subjects who underwent magnetic resonance cholangiopancreatography who were hospitalized for suspicious viral hepatic disorders and biliary disease. Of the total 409 subjects enrolled, 227 were excluded for the following reasons: (a) >7 day interval between MRI PDFF and abdominal US (n = 111), (b) inappropriate US (n = 21), (c) age <19 years (n = 4), (d) primary or secondary hepatic malignancy (n = 43), and (e) acute infectious disease (n = 48). Thus, a total of 182 subjects were included in our study cohort for this study (107 men [mean age, 49.2 years; age range, 20–80 years] and 75 women [mean age, 58.1 years; age range, 20–91 years]). A flow diagram of the patient selection process is shown in Fig. 1.

### Liver US examination

**Figure 2:**
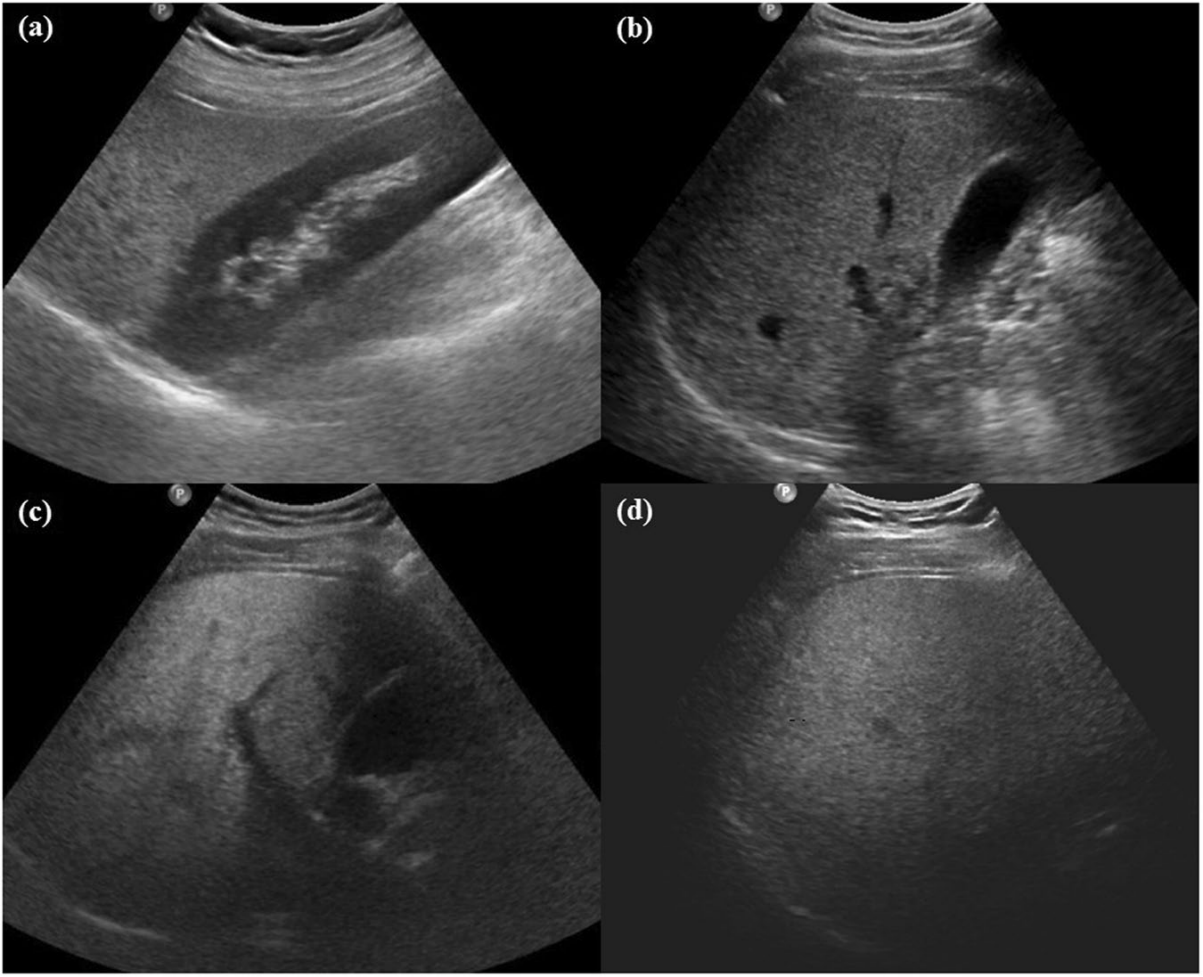
Classic examples of the four signs observed on ultrasonography. (**a**) Abnormal hepatorenal echo (liver had higher echogenicity than the right renal cortex). (**b**) Loss of echogenicity of portal vein (the echogenic wall of the main portal vein was not visible in the right intercostal view). (**c**) Posterior beam attenuation (impaired visualization of more than one-third of the hepatic parenchyma in the right intercostal view). (**d**) Poor diaphragm visualization (impaired visualization of more than half of the diaphragm in the right intercostal view).

All US examinations were performed using a standardized scanning protocol on a Philips iU22 (Philips Healthcare, Andover, MA, USA) or Aixplorer (SuperSonic Imagine, Aix-en-Provence, France) scanner with a low frequency convex transducer. Two radiologists (B.K.K and M.K, who had 9 and 6 years of experience in abdominal imaging, respectively) independently assessed the US imaging using a picture archiving and communication system (PiView, Infinitt Co., Seoul, Korea). The four US signs (abnormal hepatorenal echoes, loss of echogenicity of the portal vein, poor diaphragm visualization, and posterior beam attenuation) were evaluated to diagnose and determine the severity of fatty liver^19–21^. These observers were blinded to the clinical and histopathological data prior to analysis. On US, an abnormal hepatorenal echo was identified when the liver had higher echogenicity than the right renal cortex, loss of echogenicity of the portal vein was identified when the echogenic wall of the main portal vein was not visible in the right intercostal view, posterior beam attenuation was defined as impaired visualization of more than one-third of the hepatic parenchyma, and poor diaphragm visualization was defined as impaired visualization of more than half of the diaphragm in the right intercostal view (Fig. 2). After each radiologist had analyzed the images, a consensus was reached via discussion if they had differing opinions. Any discrepancy between the two observers regarding the four US signs was used for interobserver agreement analysis.

### MRI examination

A 3T MRI scanner (Ingenia; Philips Healthcare, Best, The Netherlands) with a torso coil was used in all MRI examinations. A three-plane localization imaging gradient echo (GRE) sequence was obtained first, followed by a 3D multiple echo GRE sequence based on the mDIXON technique (mDixon-Quant, Philips Medical systems, Best, The Netherlands) performed in a single breath-hold. The mDIXON-Quant sequence used the following parameters: six TEs (first TE 0.98 msec, delta TE 0.8 msec) and TR 6.3 msec, flip angle 3°, parallel imaging SENSE factor 2, number of signal average 1, matrix size 300 × 300, field-of-view 350 × 350 mm, number of slices 60, and slice thickness 3 mm (50% interpolation). We used a very low spin flip angle to avoid T1 saturation and 6 echo acquisition and 7 peak fat modeling for overcome complexity of fat and T2* bias. This sequence automatically generated water, fat, fat fraction, R2* and T2* maps.

### Measurement of MRI PDFF

Three non-overlapping circular ROIs of 100-mm^2^ area within each Couinaud liver segment were defined avoiding large vessels, ducts, organ boundaries, focal hepatic lesions, and imaging artifacts. A total of 24 ROIs per patient were obtained from eight segments, and the average of all measurements was defined as the mean PDFF.

### Reference standard of fatty liver

Fatty liver was graded according to the following criteria^7,22,23^: Grade 0: PDFF less than 6.4%, Grade 1: PDFF equal to or greater than 6.4% and less than 16.3%, Grade 2: PDFF equal to or greater than 16.3% and less than 21.7%, and Grade 3: PDFF equal to or greater than 21.7%.

### Statistical analyses

Baseline characteristics were calculated using descriptive statistics. Normality of the distribution was evaluated using a Shapiro-Wilk test. If the data was normally distributed, it was described as mean and standard deviation (S.D.). Mean MRI PDFF, of each US sign, and their combinations were calculated. To correlate the combinations of US signs with the mean MRI PDFF, the Spearman’s correlation coefficient was calculated with box-and-whisker plots and simple correlation analysis. The sensitivity, specificity, positive predictive value (PPV), negative predictive value (NPV), and areas under the receiver operating characteristic (ROC) curve (AUROC) with 95% confidence intervals were calculated using ROC curve analysis to evaluate the ability of US signs (alone and in combination) to predict the presence or degree of HS. In addition, we evaluated whether the diagnostic performance in detection of HS was different according to different previously published MRI PDFF cutoff values. Interobserver agreement for each US sign was analyzed using Cohen’s kappa (κ) statistics as follows: < 0.20, poor; 0.21–0.40, fair; 0.41–0.60, moderate; 0.61–0.80, good; and 0.81–1.00 was considered excellent reliability. *p* < 0.05 were considered statistically significant. We used SPSS Version 21.0 (SPSS Chicago, IL, USA) and Medcalc for Windows (Version 14.12.0; MedCalc software, Mariakerke, Belgium) for statistical analyses.

### Data availability

The datasets generated during and/or analyzed during the current study are not publicly available because of personal information protection but are available from the corresponding author on reasonable request. Technical appendix, statistical code, and dataset are available from the corresponding author at HYPERLINK “mailto:noshin@hanyang.ac.kr” noshin@hanyang.ac.kr. All relevant data are within this paper and its Supporting Information files.

## Results

In our study, the prevalence of fatty liver (MRI PDFF ≥ 6.5%) was 32.4% (59/182). Among these subjects, 31 (17.0%), 12 (6.6%), and 16 (8.8%) were classified as having grade 1, 2, and 3 steatosis according to the MRI PDFF. Among the baseline characteristics, body mass index (*p* < 0.001), and levels of triglycerides (*p* < 0.001), cholesterol (*p* < 0.001), glucose (*p* = 0.005), aspartate aminotransferase (AST) (*p* < 0.001), alanine transaminase (ALT) (*p* < 0.001), gamma-glutamyltransferase (GGT) (*p* = 0.017), and white blood cell (WBC) (*p* = 0.012) tended to increase as HS grade increased. Detailed baseline and clinical characteristics according to severity of fatty liver are shown in Table 1.

**Table 1:** Baseline characteristics according to degree of fatty liver.

Variables	Normal	Grade 1 steatosis	Grade 2 steatosis	Grade 3 steatosis	*P* value	*P* value for trend
	(PDFF < 6.5%)	(6.5% ≤ PDFF < 16.3%)	(16.3% ≤ PDFF < 21.7%)	(PDFF ≥ 21.7%)		
N (%)	123 (67.6)	31 (17.0)	12 (6.6)	16 (8.8)	…	…
Mean PDFF (%)	2.8 ± 1.3	10.1 ± 2.9	18.2 ± 1.4	29.9 ± 7.3		<0.001^†^
Age (year)	56.1 ± 15.8	52.4 ± 15.0	45.0 ± 16.7	34.8 ± 13.2	<0.001^*^	<0.001^†^
Male: Female	65:58	23:8	7:5	12:4	…	…
Body mass index (kg/ m²)	24.2 ± 3.9	27.6 ± 3.1	32.8 ± 7.8	30.6 ± 4.4	<0.001^*^	<0.001^†^
Triglycerides (mg/dL)	114.5 ± 67.0	189.7 ± 152.8	169.1 ± 84.1	159.8 ± 70.7	<0.001^*^	<0.001^†^
Cholesterol (mg/dL)	173.8 ± 41.3	194.0 ± 159.4	191.5 ± 39.5	181.2 ± 44.4	<0.001^*^	0.001^†^
Glucose (mg/dL)	113.5 ± 44.8	124.3 ± 42.5	106.9 ± 19.3	123.2 ± 29.1	0.025^*^	0.005^†^
AST (U/L)	60.5 ± 101.1	81.7 ± 102.7	75.8 ± 54.4	93.7 ± 67.8	<0.001^*^	<0.001^†^
ALT (U/L)	63.2 ± 96.1	104.0 ± 164.4	88.7 ± 76.8	137.1 ± 67.7	<0.001^*^	<0.001^†^
ALP (mg/dL)	97.2 ± 90.8	85.2 ± 37.9	76.6 ± 30.8	80.9 ± 29.6	0.827	0.486
GGT (U/L)	144.8 ± 226.6	344.0 ± 487.4	58.7 ± 36.6	157.6 ± 222.6	0.008^*^	0.017^†^
BUN (mg/dL)	14.1 ± 5.5	14.4 ± 3.3	12.8 ± 5.2	13.0 ± 4.8	0.379	0.775
Creatinine (mg/dL)	0.84 ± 0.22	0.91 ± 0.17	0.87 ± 0.12	0.87 ± 0.12	0.334	0.101
Bilirubin	1.5 ± 2.9	1.1 ± 1.4	1.6 ± 2.3	1.5 ± 2.9	0.878	0.953
WBC (/mm^3^)	6396 ± 2798	6752 ± 2648	7033 ± 2192	7481 ± 2065	0.068	0.012^†^
Platelets (/mm^3^ × 1000)	214.6 ± 78.8	200.2 ± 59.0	221.9 ± 50.8	240.4 ± 53.0	0.339	0.213
**US signs**						
Abnormal hepatorenal echoes	31 (25.2)	29 (93.5)	10 (100.0)	15 (100.0)	…	<0.001^‡^
Loss of echogenicity of portal vein	4 (3.3)	15 (48.4)	10 (100.0)	15 (100.0)	…	<0.001^‡^
Posterior beam attenuation	0	3 (9.7)	6 (50.0)	13 (81.3)	…	<0.001^‡^
Poor diaphragm visualization	0	1 (3.2)	2 (16.7)	8 (50.0)	…	<0.001^‡^

Note - Data are presented as the mean with standard deviation or as number of subjects with range or percentage in parentheses. AST, aspartate aminotransferase; ALT, alanine transaminase; ALP, alkaline phosphatase; GGT, gamma-glutamyltransferase; BUN, blood urea nitrogen; WBC, white blood cell. *p < 0.05 by ANOVA or Kruskal-Wallis test. ^†^*p* < 0.05 by Spearson correlation analysis. ^‡^*p* < 0.05 linear by linear association.

The mean MRI PDFF of each individual US sign (abnormal hepatorenal echoes, loss of echogenicity of the portal vein, posterior beam attenuation, and poor diaphragm visualization) was 12.6% (S.D. ± 10.12), 18.8% (S.D. ± 9.97), 24.0% (S.D. ± 7.60) and 25.9% (S.D. ± 7.40), respectively.

**Figure 3:**
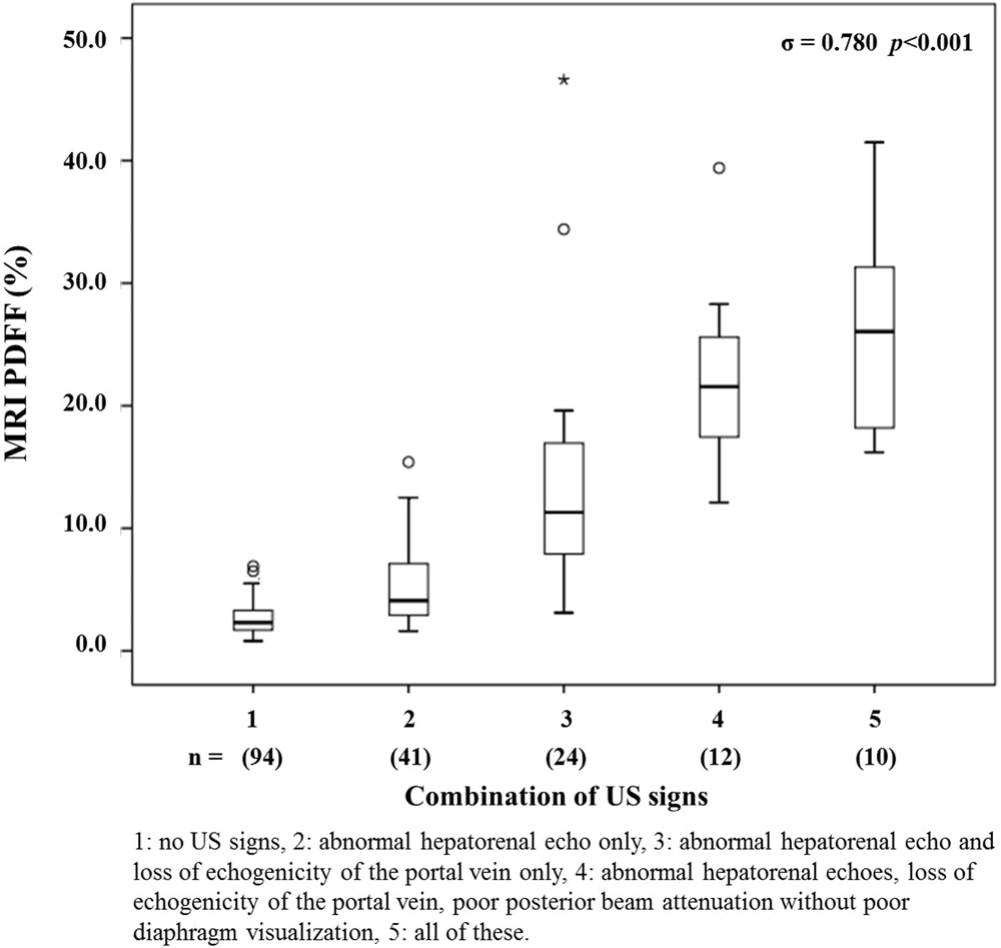
Correlation between the combinations of US signs and the magnetic resonance imaging proton density fat fraction.

In subjects without any US signs, the PDFF was 2.6% (S.D. ± 1.22). When only abnormal hepatorenal echoes were found, the PDFF was 5.4% (S.D. ± 3.35); when abnormal hepatorenal echoes and loss of echogenicity of the portal vein were present but poor diaphragm visualization and posterior beam attenuation were absent; the PDFF was 13.8% (S.D. ± 9.67). When abnormal hepatorenal echoes, loss of echogenicity of the portal vein, and poor posterior beam attenuation were visible, yet the diaphragm could not be visualized, the PDFF was 22.2% (S.D. ± 7.29). When all four US signs were seen simultaneously, the PDFF was 26.2% (S.D. ± 7.75). These combinations were named from 1 to 5 in order; there was a strong positive correlation between these signs and the MRI PDFF (σ = 0.780, *p* < 0.001) (Fig. 3).

The sensitivity and NPV for the prediction of the presence of HS were as follows: 96.6% (57/59) and 97.9% (92/94) for abnormal hepatorenal echoes, 72.9% (43/59) and 88.2% (119/135) for loss of echogenicity of the portal vein, 37.9% (22/59) and 76.9% (123/160) for posterior beam attenuation, 18.9% (11/59) and 71.9% (123/171) for poor diaphragm visualization (Table 2).

**Table 2:** Diagnostic performance of each ultrasonography sign for diagnosis of fatty liver.

US signs	Severity of FL	SN	SP	PPV	NPV	AUC (95% CI)
Abnormal hepatorenal echoes	≥Grade 1 (PDFF ≥ 6.4%)	96.6 (57/59)	74.8 (92/123)	64.8 (57/88)	97.9 (92/94)	0.857 (0.798–0.904)
	≥Grade 2 (PDFF ≥ 16.3%)	100.0 (28/28)	61.0 (94/154)	31.8 (28/88)	100.0 (94/94)	0.805 (0.740–0.860)
	≥Grade 3 (PDFF ≥ 21.7%)	100.0 (16/16)	56.6 (94/166)	18.2 (16/88)	100.0 (94/94)	0.783 (0.716–0.841)
Loss of echogenicity of the portal vein	≥Grade 1 (PDFF ≥ 6.4%)	72.9 (43/59)	96.8 (119/123)	91.5 (43/47)	88.2 (119/135)	0.846 (0.785–0.895)
	≥Grade 2 (PDFF ≥ 16.3%)	100.0 (28/28)	87.7 (135/154)	59.6 (28/47)	100.0 (135/135)	0.938 (0.893–0.969)
	≥Grade 3 (PDFF ≥ 21.7%)	100.0 (1/16)	81.3 (135/166)	34.0 (16/47)	100.0 (135/135)	0.907 (0.855–0.945)
Posterior beam attenuation	≥Grade 1 (PDFF ≥ 6.4%)	37.9 (22/59)	100.0 (123/123)	100.0 (22/22)	76.9 (123/160)	0.690 (0.617–0.756)
	≥Grade 2 (PDFF ≥ 16.3%)	67.9 (19/28)	98.1 (151/154)	86.4 (19/22)	94.4 (151/160)	0.830 (0.767–0.881)
	≥Grade 3 (PDFF ≥ 21.7%)	81.3 (13/16)	94.6 (157/166)	59.1 (13/22)	98.1 (157/160)	0.879 (0.823–0.923)
Poor diaphragm visualization	≥Grade 1 (PDFF ≥ 6.4%)	18.9 (11/59)	100.0 (123/123)	100.0 (11/11)	71.9 (123/171)	0.595 (0.519–0.667)
	≥Grade 2 (PDFF ≥ 16.3%)	35.7 (10/28)	99.4 (153/154)	90.9 (10/11)	89.5 (153/171)	0.675 (0.602–0.743)
	≥Grade 3 (PDFF ≥ 21.7%)	50.0 (8/16)	98.2 (163/166)	72.7 (8/11)	95.3 (163/171)	0.741 (0.671–0.803)

Note - SN, sensitivity; SP, specificity; PPV, positive predictive value; NPV, negative predictive value; AUC, area under the curve; CI, confidence interval.

In the combinations of US signs, abnormal hepatorenal echoes with loss of echogenicity of the portal vein showed the highest sensitivity of 72.9% (43/59) and NPV 88.2% (119/135) for the presence of fatty liver without much difference of specificity and PPV compared with other combinations of US signs (Table 3). However, the sensitivity and NPV were not higher than for the abnormal hepatorenal echo alone.

**Table 3:** Diagnostic performance of combinations of ultrasonography signs for presence of fatty liver (PDFF ≥ 6.4%).

US signs	SN	SP	PPV	NPV	AUC (95% CI)
Abnormal hepatorenal echoes	96.6 (57/59)	74.8 (92/123)	64.8 (57/88)	97.9 (92/94)	0.857 (0.798–0.904)
Loss of echogenicity of the portal vein	72.9 (43/59)	96.8 (119/123)	91.5 (43/47)	88.2 (119/135)	0.846 (0.785–0.895)
Posterior beam attenuation	37.9 (22/59)	100.0 (123/123)	100.0 (22/22)	76.9 (123/160)	0.690 (0.617–0.756)
Poor diaphragm visualization	18.9 (11/59)	100.0 (123/123)	100.0 (11/11)	71.9 (123/171)	0.595 (0.519–0.667)
Liver echo + Loss of portal vein	72.9 (43/59)	96.8 (119/123)	91.5 (43/47)	88.2 (119/135)	0.848 (0.788–0.897)
Liver echo + Posterior attenuation	37.3 (22/59)	100.0 (123/123)	100 (22/22)	76.9 (123/160)	0.686 (0.614–0.753)
Liver echo + Poor diaphragm	18.6 (11/59)	100.0 (123/123)	100 (11/11)	71.9 (123/171)	0.593 (0.518–0.665)
Loss of portal vein + Posterior attenuation	37.3 (22/59)	100.0 (123/123)	100 (22/22)	76.9 (123/160)	0.686 (0.614–0.753)
Loss of portal vein + Poor diaphragm	18.6 (11/59)	100.0 (123/123)	100 (11/11)	71.9 (123/171)	0.593 (0.518–0.665)
Posterior attenuation + Poor diaphragm	16.9 (10/59)	100 (123/123)	100 (10/10)	71.5 (123/172)	0.585 (0.510–0.657)
Liver echo + Loss of portal vein + Posterior attenuation	37.3 (22/59)	100.0 (123/123)	100 (22/22)	76.9 (123/160)	0.686 (0.614–0.753)
Liver echo + Loss of portal vein + Poor diaphragm	18.6 (11/59)	100.0 (123/123)	100 (11/11)	71.9 (123/171)	0.593 (0.518–0.665)
Liver echo + Posterior attenuation + Poor diaphragm	16.9 (10/59)	100 (123/123)	100 (10/10)	71.5 (123/172)	0.585 (0.510–0.657)
Loss of portal vein + Posterior attenuation + Poor diaphragm	16.9 (10/59)	100 (123/123)	100 (10/10)	71.5 (123/172)	0.585 (0.510–0.657)
Liver echo + Loss of portal vein + Posterior attenuation + Poor diaphragm	16.9 (10/59)	100 (123/123)	100 (10/10)	71.5 (123/172)	0.585 (0.510–0.657)

Note - SN, sensitivity; SP, specificity; PPV, positive predictive value; NPV, negative predictive value; AUC, area under the curve; CI, confidence interval; Liver echo, Abnormal hepatorenal echoes; Loss of portal vein, Loss of echogenicity of the portal vein; posterior attenuation, Posterior beam attenuation; Poor diaphragm, Poor diaphragm visualization.

We then simulated diagnostic performance of abnormal hepatorenal echoes according to previously published thresholds^24,25^ (Table 4). Each of two previously published cutoff points distinguished steatosis grades 0 and 1. The sensitivity and NPV to predict the presence of fatty liver were as follows: 80.2% (73/59) and 80.9% (76/97) for the 3.5% cutoff, and 92.7% (63/68) and 94.7% (89/94) for the 5.2% cutoff.

**Table 4:** Diagnostic accuracy of abnormal hepatorenal echoes according to proposed thresholds for presence of fatty liver.

Author	Imajo^24^	Martino^25^	Tang^22,23^
Cutoff	3.5	5.2	6.4
SN	80.2 (73/91)	92.7 (63/68)	96.6 (57/59)
SP	83.5 (76/91)	78.1 (89/114)	74.8 (92/123)
PPV	83.0 (73/88)	71.6 (63/88)	64.8 (57/88)
NPV	80.9 (76/94)	94.7 (89/94)	97.9 (92/94)
AUC (95% CI)	0.819 (0.755–0.872)	0.854 (0.794–0.902)	0.857 (0.798–0.904)

Note - FL, fatty liver; SN, sensitivity; SP, specificity; PPV, positive predictive value; NPV, negative predictive value; AUC, area under the curve; CI, confidence interval.

In the presence of abnormal echo and loss of echogenicity of the portal vein, sensitivity and specificity in detecting steatosis grade 2 or higher was 100% (28/28) and 87.7% (135/154). In the presence of all 4 US signs, sensitivity and specificity in detecting grade 3 steatosis was 43.8% (7/16) and 98.2% (163/166) (Table 5).

**Table 5:** Diagnostic performance of combinations of ultrasonography signs for predicting degree of fatty liver.

Severity of FL	Combinations of US Findings	SN	SP	PPV	NPV	AUC (95% CI)
Grade 0 (<6.4%)	None	74.8 (92/123)	96.6 (57/59)	97.9 (92/94)	64.8 (57/88)	0.8575 (0.797–0.904)
≥Grade 1 (PDFF ≥ 6.4%)	Liver echo	96.6 (57/79)	74.8 (92/123)	64.8 (57/88)	97.9 (92/94)	0.857 (0.797–0.904)
≥Grade 2 (PDFF ≥ 16.3%)	+Loss of portal vein	100.0 (28/28)	87.7(135/154)	59.6 (28/47)	100.0 (135/135)	0.938 (0.893–0.969)
≥Grade 3 (PDFF ≥ 21.7%)	+Posterior attenuation	81.3 (13/16)	94.6 (157/166)	59.1 (13/22)	98.1 (157/160)	0.879 (0.823–0.923)
≥Grade 3 (PDFF ≥ 21.7%)	+Poor diaphragm	43.8 (7/16)	98.2 (163/166)	70.0 (7/10)	94.8 (163/172)	0.710 (0.638–0.774)

Note - FL, fatty liver; SN, sensitivity; SP, specificity; PPV, positive predictive value; NPV, negative predictive value; AUC, area under the curve; CI, confidence interval; Liver echo, abnormal hepatorenal echo only; +Loss of portal vein, abnormal hepatorenal echo and loss of echogenicity of the portal vein; +Posterior attenuation, abnormal hepatorenal echo and loss of echogenicity of the portal vein and poor posterior beam attenuation; +Poor diaphragm, abnormal hepatorenal echo and loss of echogenicity of the portal vein and poor posterior beam attenuation and poor diaphragm visualization.

The two US readers showed excellent agreement for loss of echogenicity of the portal vein (κ = 0.859) and good agreement for abnormal hepatorenal echoes (κ = 0.759), poor diaphragm visualization (κ = 0.767), and posterior beam attenuation (κ = 0.776) (Table 6).

**Table 6:** Interobserver agreement of ultrasonography signs.

US signs	Interobserver agreement	
	κ value (95% CI)	*P* value
Abnormal hepatorenal echoes	0.759 (0.665–0.852)	<0.001
Loss of echogenicity of the portal vein	0.859 (0.774–0.944)	<0.001
Posterior beam attenuation	0.776 (0.626–0.925)	<0.001
Poor diaphragm visualization	0.767 (0.547–0.987)	<0.001

Note – CI, confidence interval.

## Discussion

Conventional US signs of HS have been well described in the literature^19–21^ but they have not been compared to MRI PDFF. We found positive correlations between some combinations of US signs, and MRI PDFF values. In addition, the ability of ‘abnormal hepatorenal echoes’ to predict the presence of fatty liver (≥6.5% MRI PDFF) was high with a sensitivity of 96.6% and NPV of 97.9%, respectively. In a meta-analysis that compared the diagnostic performance of US for fatty liver, the diagnostic performance of US to detect a histologic steatosis grade 2 or higher was excellent, with a sensitivity of 85.7% and a specificity of 85.2%^17^. However, the diagnostic performance of US for histologic grade 1 steatosis was relatively low at 12.0–49.8%^15,16^. Previous studies have found that the sensitivity and specificity for detecting less than histologic 10% fatty liver were 73.3% and 84.4%, respectively^17,18^. Another previous study reported that the sensitivity was merely 12% in subjects with a histologic hepatic fat content of 5–10%^16^. Even though it is difficult to make direct comparisons of the diagnostic performance because of the difference reference standards used, this study demonstrated that the sensitivity and NPV of ‘abnormal hepatorenal echoes’ in detecting steatosis grade 1 or higher was good with a sensitivity of 80.2–96.6% and a NPV of 80.9–97.9% according to the threshold used in this study as well as with previously published thresholds.

Prior reports of US diagnoses of fatty liver have been largely subjective^26,27^. In our study, we attempted to improve the objectivity of US assessment of HS. Abnormal hepatorenal echoes were observed in 25.2% of subjects (31/123) without fatty liver on MRI PDFF. This suggests that the NPV to diagnose fatty liver using abnormal hepatorenal echoes was high, while the PPV was low. High sensitivity and NPV are important for screening given their utility when ruling out fatty liver disease. NAFLD is most common cause of unexplained liver enzyme elevation^28^. Ruling out fatty liver disease using US is very helpful in routine clinical practice for a physician examining patients with elevated liver enzymes. Moreover, it is very important to define healthy controls. NAFLD is common, but often missed in volunteers for clinical trials, despite its potential effect on subject safety and validity of results^29^. Increasing awareness of NAFLD prevalence and ruling out NAFLD using US may ameliorate this problem.

A possible reason for the increased sensitivity of US in detecting grade 1 or higher steatosis compared to previous studies may be related to the severity and distribution of fatty liver in the patient cohort. In a previous study with a large number of liver transplantation donors, the sensitivity of US was low in subjects with fatty liver less than or equal to 30% by histology^15,16^, and other subsequent studies reported that sensitivity was also low in those with fatty liver less than or equal to 20% and 12.5% by histology, respectively^19,30^. Considering that mild fatty liver is a broad spectrum that consists of 5–33% macrosteatosis, different results may be obtained based on the distribution of fat fraction of the subjects. In fact, the high sensitivity of our study might likely be attributable to the higher threshold of fatty liver detected by MRI PDFF (6.5%) than in previous studies^24,25^. However, the threshold used in our study was reconfirmed as having moderate to high sensitivity and high specificity in a recent study carried out by the same researchers who compared liver biopsy and MRI PDFF in an independent cohort^22^.

The severity of fatty liver has been evaluated qualitatively as normal, mild, moderate, and severe using US signs of echogenicity of the portal vein, poor diaphragm visualization, and posterior beam attenuation^20,31,32^. We found that when only abnormal hepatorenal echoes and loss of echogenicity of the portal vein were observed with no diaphragm visualization or posterior beam attenuation, the MRI PDFF was 13.8% (S.D. ± 9.67), which was close to the value of grade 2 steatosis determined by MRI PDFF^6,7,22,23^. When an abnormal hepatorenal echoes and loss of echogenicity of the portal vein were visible, the sensitivity and specificity in detecting grade 2 or higher steatosis were 100% and 85.9%, respectively, with an AUROC of 0.930. These results of our study were comparable to those of a previous prospective study^19^. Furthermore, the diagnostic sensitivity and specificity for grade 3 steatosis was good when considering posterior beam attenuation.

To overcome the limitation of conventional US with its qualitative nature, quantitative ultrasound (QUS) was developed to characterize tissue microstructure objectively. QUS is a technique that can assess HS by measuring fundamental liver tissue parameters, including attenuation coefficient and backscatter coefficient (BSC)^33,34^. Although QUS is useful for grading HS, it also has limited availability in most smaller clinical trials because it requires post-processing software and cannot be evaluated in real time. In our study, we attempted to compare MRI PDFF with conventional US signs; thus, no comparison with QUS was performed. Future prospective studies are needed to compare combinations of conventional US signs with QUS, MRI PDFF, and liver biopsy in a large cohort of patients for grading of HS.

There were several limitations to our study. First, conventional US signs may be operator-dependent and subjective, although the interobserver agreement in our study was good to excellent (κ value: 0.759–0.858) for evaluation of US signs measured independently by two radiologists. This may be because this study did not qualitatively evaluate the severity of fatty liver, as in previous reports^31^, but evaluated the agreement of each US sign independently. Our results may be promising, but must be validated prospectively in multi-center, community-based clinical trials before MRI PDFF can be adopted in a routine clinical setting. Second, the the reference standard for evaluating HS was MRI PDFF. The invasive gold standard of diagnosis of fatty liver is biopsy, but it is an invasive approach and there are concerns about its accuracy because of sampling bias and poor interobserver correlations^4,35^. Recent studies have reported that MRI PDFF can measure hepatic fat content more objectively and accurately than biopsy^25,36^. However, the cutoff value of fatty liver by MRI PDFF differs depending on the researcher. Therefore, this study was performed using the most recent and most verified results. Third, the percentage of subjects with moderate or severe fatty liver included in this study was relatively low, at 15.4%. Finally, US signs for longitudinal follow up of HS were not evaluated in this study, additional larger studies are needed to define these US signs in a longitudinal setting.

In conclusion, the sensitivity and NPV for the determination of HS by US using MRI PDFF as a reference standard were good at 96.6% and 97.7%, respectively, and US may be considered a suitable screening tool for the exclusion of fatty liver.

## Electronic supplementary material

Appendix1
