# Supplementary material for: Comparison of conventional sonographic signs and magnetic resonance imaging proton density fat fraction for assessment of hepatic steatosis
Source: Sci Rep. 2018 May 17;8:7759. doi: 10.1038/s41598-018-26019-x (PMC5958077; doi:10.1038/s41598-018-26019-x)
Supplement: Supplementary file 1 — Appendix1 [file 41598_2018_26019_MOESM1_ESM.docx]

**Comparison of conventional sonographic signs and magnetic resonance imaging proton density fat fraction for assessment of hepatic steatosis**

Mimi Kim^1^, Bo-Kyeong Kang^1*^, and Dae Won Jun^2*^

**Appendix 1**

Inclusion criteria were (a) subjects aged 19 to 75, (b) among the subjects suffered liver disease for 3months, those who have abnormal liver function test (45U/L≤ALT≤225U/L) within the last 2 weeks at the screening visit and (c) subjects who signs the clinical trial agreement.

Exclusion criteria were (a) subjects allergic to the experimental medicine, (b) subjects with any liver disease affecting the outcome of clinical trials (however, HBV or HCV carriers who are not taking antiviral drugs could be enrolled in the study by the researcher judgment), (c) decompensated liver cirrhosis (Child-Pugh grade B or C), (d) subjects who has been treated with chemotherapy or radiation therapy for liver cancer or any malignant tumors within 6 months, (e) subjects taking psychiatric medicine for major depression, (f) subjects with active bleeding, (g) subjects with acute bacterial infection, (h)subjects who have experienced the following severe conditions within the last 6 months: Coronary artery bypass surgery, angioplasty, vascular stent, Acute myocardial infarction, Angina, Congestive heart failure, Uncontrolled hypertension (BP>180/110mmHg despite maximum drug management), Uncontrolled ventricular arrhythmia or ventricle requiring continuous therapy, Arrhythmia, Severe biliary atresia (jaundice level> 3 mg/dL), (i) subjects suffering from severe systemic disease that can interfere with daily living (Obstructive pulmonary disease, etc), (j) pregnant women or lactating women (in case of fertile women, those who are not willing to use effective methods of contraception during the trial period), (k) subjects who participated in other clinical trials within 3 months, (l) poor compliant subjects and (m) illiterate subjects
